# Positron-emission tomography as a predictor of response to first-line cyclin-dependent kinase 4/6 inhibitors in patients with metastatic ER-positive HER2-negative breast cancer

**DOI:** 10.1038/s41523-026-00962-9

**Published:** 2026-05-11

**Authors:** Marcin Kubeczko, Anna Polakiewicz-Gilowska, Andrea d’Amico, Olgierd Chrabański, Katarzyna Świderska, Ewa Chmielik, Barbara Bobek-Billewicz, Daria Handkiewicz-Junak, Michał Jarząb

**Affiliations:** 1 Breast Cancer Center, Maria Sklodowska-Curie National Research Institute of Oncology, Gliwice, Poland; 2 Department of Nuclear Medicine and Endocrine Oncology, Maria Sklodowska-Curie National Research Institute of Oncology, Gliwice, Poland; 3 Tumor Pathology Department, Maria Sklodowska-Curie National Research Institute of Oncology, Gliwice, Poland; 4 Radiology and Diagnostic Imaging Department, Maria Sklodowska-Curie National Research Institute of Oncology, Gliwice, Poland

**Keywords:** Breast cancer, Targeted therapies

## Abstract

Cyclin-dependent kinase 4/6 inhibitors (CDK4/6i) are the standard first-line treatment for metastatic estrogen receptor–positive, HER2-negative breast cancer, yet acquired resistance remains a major challenge. Reliable biomarkers for prognostic stratification are therefore needed. We retrospectively analyzed 174 patients treated with first-line CDK4/6i between 2018 and 2023 who underwent baseline 18F-fluorodeoxyglucose positron emission tomography. Associations between baseline SUVmax and clinical outcomes were assessed using Kaplan–Meier estimates, Cox proportional hazards models, logistic regression for early progression, and receiver-operating characteristic (ROC) analysis for cut-off determination. Higher SUVmax was significantly associated with shorter progression-free survival (PFS; HR 1.11, 95% CI 1.05–1.16, *p* < 0.001) and overall survival (OS; HR 1.10, 95% CI 1.02–1.18, *p* = 0.016). Patients with SUVmax <8.0 experienced markedly longer PFS and improved 2-year PFS compared with those with SUVmax ≥8.0. Similarly, 2-year OS was substantially higher among patients with lower SUVmax values. In multivariable analysis, SUVmax remained an independent prognostic factor for PFS. Higher SUVmax was also associated with early progression, with an 18% increase in odds per unit (OR 1.18, 95% CI 1.05–1.33). These findings demonstrate that baseline SUVmax is a strong and readily accessible prognostic biomarker in patients receiving CDK4/6i, helping to identify individuals at elevated risk of early disease progression. Prospective validation is warranted.

## Introduction

Breast cancer is an authentic global health challenge that has an immense medical need, demanding effective interventions and personalized therapies^1,2^. However, not all patients respond uniformly to treatment due to the disease’s heterogeneity^2^. In this context, research aimed at patient stratification and therapy personalization becomes paramount^3–5^.

Cyclin-dependent kinase 4/6 inhibitors (CDK4/6i) are the standard-of-care in the treatment of advanced estrogen receptor (ER)-positive human epidermal growth factor 2 receptor (HER2)-negative breast cancer^6^. In addition to prolonging progression-free survival (PFS) and overall survival (OS), quality of life (QoL) is also improved^7^. Their efficacy extends to both de novo and recurrent metastatic breast cancer^8^. However, acquired resistance further complicates advanced breast cancer management^3^. Identifying patients who derive limited benefit from CDK4/6i combined with endocrine treatment is crucial. While liquid biopsy holds promise^4^, imaging studies remain the cornerstone of assessment^5^.

In recent years, ^18^F-FDG PET/CT has gained increasing attention as a quantitative imaging biomarker in breast cancer. According to the recent EANM/SNMMI joint guideline, metabolic PET parameters—including standardized uptake value (SUV), metabolic tumor volume (MTV), and total lesion glycolysis (TLG)—are associated with tumor aggressiveness and may have prognostic value in both early and metastatic settings^9^. SUV-based metrics are particularly attractive because they are routinely reported in clinical PET/CT examinations, standardized through EARL (EANM Research GmbH) accreditation, and require no additional post-processing. However, while several studies have explored metabolic biomarkers in metastatic breast cancer, evidence specifically addressing patients treated with CDK4/6i remains limited.

Accurate baseline imaging is essential in metastatic breast cancer (MBC), as treatment evaluation depends on the initial disease burden^5,10^. While contrast-enhanced computed tomography remains the standard staging modality in clinical practice and trials^11–14^, molecular imaging techniques are gaining increasing prominence^15^.

^18^F-fluorodeoxyglucose (^18^F-FDG) PET/CT is an established molecular imaging technique in breast cancer, widely used for staging and detection of recurrence^16,17^. Compared with contrast-enhanced CT (CE-CT) ^18^F-FDG-PET/CT more frequently detects bone and distant lymph node metastases, while CE-CT is more sensitive for liver and lung lesions; concordance is highest for bone and liver and lowest for lymph nodes^18^. Tumor histopathology and biology influence ^18^F-FDG uptake, with lower uptake typically seen in lobular and low-grade cancers^19,20^. Several studies have examined associations between FDG avidity and tumor type, grade, and receptor status, although findings have been inconsistent and largely derived from early breast cancer cohorts^21–23^.

The range of radiotracers for molecular imaging in breast cancer is expanding^24^. ^18^F-fluoroestradiol (^18^F-FES) allows whole-body ER expression assessment and may identify a subset of patients who benefit from letrozole combined with CDK4/6i, with potential value both at initial diagnosis of MBC and after progression on endocrine therapy^25,26^. However, ^18^F-FES shows lower sensitivity than ^18^F-FDG PET/CT in ER-positive disease recurrence^27^ and cannot be used for staging^28^, whereas ^18^F-FDG PET/CT may serve as a comprehensive staging modality in MBC^29^.

Four pioneering studies have been published to date regarding patients with HR-positive HER2-negative MBC treated with CDK4/6i in combination with endocrine therapy. These studies comprised a total of 65 patients^30–33^. Recently, results of a larger cohort of 114 patients were published in this clinical setting^34^. Various methodologies were used in these studies, including the European Organization for Research and Treatment of Cancer (EORTC), Positron Emission Tomography Response Criteria in Solid Tumors (PERCIST), whole-body MTV, whole-body TLG, and the Deauville score^30–34^. Some of these studies found a correlation between metabolic parameters and PFS^31,34^. However, no association with OS was found in this clinical scenario.

Importantly, the prognostic value of baseline SUV_max_ alone, despite being the most accessible PET metric, has not been fully defined in this population. This study aims to address this gap. We hypothesized that baseline metabolic activity measured by SUV_max_ is associated with treatment outcomes in ER-positive/HER2-negative MBC treated with first-line CDK4/6i, and that SUV_max_ may help identify patients at increased risk of early disease progression. Therefore, the objective of this study was to assess the prognostic value of baseline SUV_max_ for PFS and OS, and its association with early radiologic progression, in a real-world cohort of MBC patients undergoing CDK4/6i therapy.

## Results

### Baseline characteristics

176 patients treated in the first-line setting with CDK4/6i and endocrine therapy underwent baseline ^18^F-FDG-PET/CT. Among these patients, two individuals did not exhibit FDG uptake in their scans: the first patient with lobular carcinoma, Grade 2, ER 100%, PR 90%, Ki67 3%, with bone and pleural metastases, who continued CDK4/6i treatment for 20.3 months; the second patient also had bone and pleural metastases, ER 100%, PR 100%, Ki67 40%, who continued CDK4/6i treatment for 12.1 months (histopathological diagnosis was based on pleural biopsy, and histology was not further specified). The remaining 174 patients (comprising 172 females and two males) with ^18^F-FDG uptake were included in the study.

Seventy-five patients (43%) had experienced disease recurrence after radical treatment. Among these, 17 patients relapsed within the first two years of adjuvant endocrine therapy, meeting the criteria for primary endocrine resistance. Bone-only disease was observed in 93 patients (53.5%), whereas 53 patients (30.5%) had both bone and visceral metastases. Detailed information on the number and anatomical distribution of metastatic lesions, as well as additional patient characteristics, is presented in Table 1.

**Table 1:** Patients characteristics

Characteristics	*N* = 174 [%]
Age, mean (range)	61(29–80)
De novo metastatic	99 [56.9]
Recurrent disease	75 [43.1]
ECOG PS	
ECOG 0	85 [48.9]
ECOG 1	68 [39.1]
ECOG 2	21 [12.0]
Metastatic site	
Bone	146 [83.9]
Distant LN	59 [33.9]
Liver	39 [22.4]
Lung	43 [24.7]
No. of metastases	
≤5	57 [32.8]
6–10	27 [15.5]
>10	90 [51.7]
Histopathological type	
Not otherwise specified	100 [57.5]
Invasive lobular carcinoma	28 [16.1]
Other histologic type	4 [2.3]
Tumor grade	
1	5 [2.9]
2	95 [54.6]
3	22 [12.6]
Ki67, median (IQR)	20% (15–30)
CDK4/6i	
Ribociclib	110 [63.2]
Palbociclib	28 [16.1]
Abemaciclib	36 [20.7]
Endocrine Tx	
Letrozole	148 [85.1]
Fulvestrant	26 [14.9]
Follow-up, mean	22.9 months

Percents do not add to 100% in all cases due to non-reported values.

*ECOG PS* Eastern Cooperative Oncology Group performance status, *LN* lymph nodes, *No. of metastases* number of distant metastases, *CDK4/6i* cyclin-dependent kinase 4/6 inhibitors, *Tx* treatment.

SUV_max_ was derived from the single hottest lesion, irrespective of location. In 82 patients (47.1%) the lesion with the highest SUV_max_ was a bone metastasis. In 36 patients (20.7%) it was the primary breast tumor; in 18 patients (10.3%) distant lymph nodes; in 15 patients (8.6%) regional lymph nodes; in 8 patients (4.6%) liver metastases; in 10 patients (5.8%) lung metastases; and in 5 patients (2.9%) other sites. A comparison between baseline SUV_max_ and clinicopathologic factors is shown in Table 2.

**Table 2:** Comparison between baseline SUV_max_ and clinicopathologic factors

Variable		*N* = 174 (%)	Median SUV_max_ (IQR)	*p*
Age	<50	43 (24.7)	9.9 (7.4–12.9)	**0.040**
	≥50	131 (75.3)	8.3 (5.9–11.7)	
Histology	NOS	100 (57.5)	8.3 (6.2–11.8)	0.094
	ILC	28 (16.1)	6.9 (4.8–9.8)	
Grade	G1-2	100 (57.5)	8.0 (6.0–11.2)	**0.029**
	G3	22 (12.6)	10.9 (7.3–13.8)	
ER	≥90	130 (74.7)	8.5 (6.2–11.6)	0.392
	<90	20 (12.6)	9.6 (6.2–12.8)	
PR	≥25	93 (53.5)	8.5 (5.9–11.9)	0.457
	<25	57 (32.8)	9.1 (6.6–11.6)	
HER2	IHC 0	70 (40.2)	10.1 (7.4–13.4)	**0.009**
	IHC 1+ & 2+	79 (45.4)	7.3 (5.7–10.2)	
Ki67	≥ 30	51 (35.2)	10.2 (7.3–13.5)	**<0.001**
	<30	94 (64.8)	7.7 (5.7–10.2)	

Statistically significant results are in bold. Percents do not add to 100% in all cases due to non-reported values.

*N* number, *SUV*_max_ maximum standardized uptake value, *IQR* interquartile range, *NOS* not otherwise specified, *ILC* invasive lobular carcinoma, *G* grade, *ER* estrogen receptor, *PR* progesterone receptor, *HER2* human epidermal growth factor 2 receptor, *IHC* immunohistochemistry.

### Treatment efficacy

**Fig. 1:**
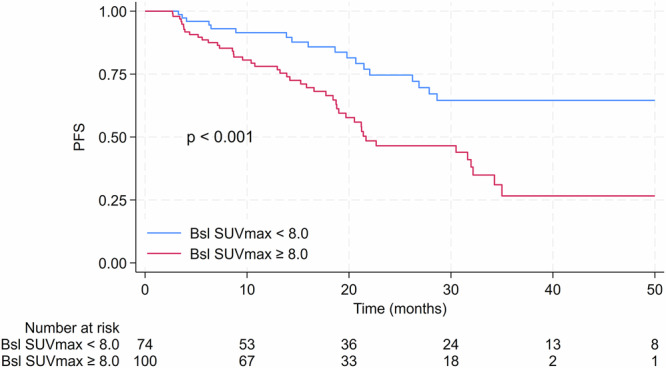
Kaplan–Meier survival estimates for progression-free survival (PFS) according to baseline SUV_max_. PFS in patients with baseline SUV_max_ <8.0 versus ≥8.0. The blue line represents patients with baseline SUV_max_ <8.0, and the red line represents patients with baseline SUV_max_ ≥8.0. PFS progression-free survival, SUV_max_ maximum standardized uptake value, Bsl baseline.

The median PFS in our study cohort was 32.0 months. The 12-month PFS was 83.8% (95% CI 77.0–88.7%), while the 24-month PFS was 58.9% (95% CI 49.4–67.2%). We observed a significant association between baseline SUV_max_ and PFS (HR 1.11; 95% CI 1.05–1.16; *p* < 0.001). Specifically, PFS was significantly improved in patients with lower baseline SUV_max_. For patients with SUV_max_ less than 8.0, the median PFS extended to 61.6 months, whereas those with SUV_max_ ≥ 8.0 experienced a median PFS of 21.7 months (24-month PFS 74.6%; 95% CI 60.4–84.4% vs. 46.5%; 95% CI 33.9–58.2%, respectively; *p* < 0.001). Kaplan–Meier survival estimates for PFS are shown in Fig. 1.

**Fig. 2:**
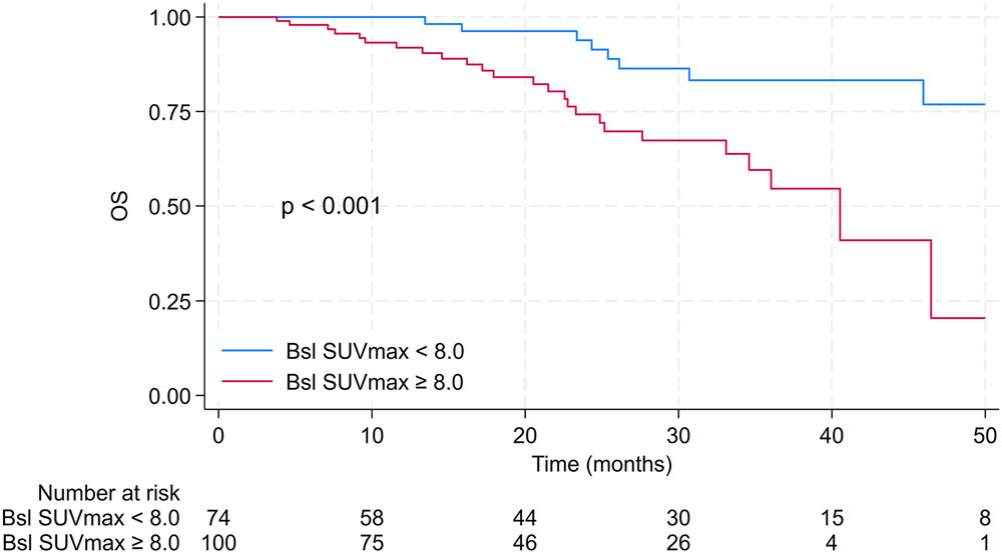
Kaplan–Meier survival estimates for overall survival (OS) according to baseline SUV_max_. OS in patients with baseline SUV_max_ <8.0 versus ≥8.0. The blue line represents patients with baseline SUV_max_ <8.0, and the red line represents patients with baseline SUV_max_ ≥8.0. OS overall survival, SUV_max_ maximum standardized uptake value, Bsl baseline.

The median OS in our study cohort was not reached. The 24-month OS was 83.0% (95% CI 74.6–88.8%), and the 36-month OS was 70.7% (95% CI 59.7–79.2%). We observed a significant association between baseline SUV_max_ and OS (HR 1.10; 95% CI 1.02–1.18, *p* = 0.016). Patients with lower baseline SUV_max_ exhibited improved OS. Among patients with SUV_max_ less than 8.0, the median OS was not reached. The 24-month was OS 93.9% (95% CI 82.0–98.0%), whereas the 36-month OS was 83.3% (95% CI 67.7–91.8%). In contrast, patients with SUV_max_ ≥ 8.0 had a median OS of 40.5 months, with the 24-month OS of 74.2% (95% CI 61.2–83.5%), and the 36-month OS of 59.5% (95% CI 43.0–72.7%). The difference between these groups was statistically significant (*p* < 0.001). Kaplan–Meier survival estimates for OS are shown in Fig. 2.

### Cut-offs calculations

In pivotal clinical trials of CDK4/6i combined with endocrine treatment in the first-line setting, PFS was 28.2 months for abemaciclib^11^, 24.8 months for palbociclib^12^, and 25.3 months for ribociclib^6^. Thus, to establish a meaningful cut-off, we selected a 24-month timepoint for our calculations. A cut-off value of 8.0 for SUV_max_ yielded 74% sensitivity and 57% specificity with an area under the receiver operating characteristic curve (AUC) of 0.65 for predicting progression before 24 months of CDK4/6i treatment. 100 patients had a baseline SUV_max_ equal to or greater than 8.0, while 74 patients had a baseline SUV_max_ less than 8.0. Baseline SUV_max_ of 8.0 or higher was significantly associated with a higher risk of progression before 24 months (OR 3.63; 95% CI 1.61–8.23; *p* = 0.002). ER of 90% or more was significantly associated with a lower risk of progression before 24 months (OR 0.15; 95% CI 0.03–0.71; *p* = 0.017). PR of 25% or more was significantly associated with a lower risk of progression before 24 months (HR 0.23; 95% CI 0.10–0.55; *p* = 0.001). The optimal calculated cut-off for Ki67 was 30%, with a sensitivity of 0.55, specificity of 0.79, and an AUC of 0.67. Patients with Ki67 ≥ 30% had a substantially higher risk of progression before 24 months, corresponding to a 4.5-fold increase in the odds of early progression (OR 4.48; 95% CI 1.77–11.30; *p* = 0.002). A weak correlation was observed between Ki67 and SUV_max_ (Spearman *ρ* = 0.32; *p* < 0.001).

### Cox regression model for the prediction of progression-free survival and overall survival

In the multivariable Cox model, baseline SUV_max_ remained an independent predictor of shorter PFS. Each 1-unit increase in SUV_max_ was associated with a 12% higher hazard of progression, while a 5-unit increase corresponded to an approximately 77% higher risk (HR ≈ 1.77). Histologic subtype was also independently associated with outcome: invasive lobular carcinoma and rare histologic subtypes had significantly better PFS compared with invasive carcinoma of no special type (NOS). Ki67, analyzed as a continuous variable, remained independently prognostic, with each 1% increase corresponding to a 3% increase in the hazard of progression. Other clinical variables—including ECOG performance status, de novo presentation, number of metastatic sites, histopathological grade, and liver involvement—were not independently associated with PFS. The multivariable Cox model for PFS included 115 patients with complete data, including histopathological subtype, tumor grade, and Ki67.

In the multivariable Cox model for OS, baseline SUV_max_ was not independently associated with outcome (HR 1.06 per unit; 95% CI 0.94–1.19; *p* = 0.375). ECOG performance status was the strongest predictor of OS, with patients presenting with ECOG 1 and 2 experiencing significantly worse survival compared with those with ECOG 0. Ki67, analyzed as a continuous variable, was also independently associated with OS, with each 1% increase corresponding to a 3% increase in the hazard of death. Tumor grade was prognostic as well, with grade 1 and 2 disease associated with more favorable survival compared with grade 3. Other clinicopathologic variables, including de novo metastatic presentation, number of metastatic sites, and primary endocrine resistance, were not independently associated with OS. The multivariable Cox model for OS included 117 patients with complete data, including Ki67 and tumor grade. Results are presented in Table 3.

**Table 3:** Cox regression models for progression-free survival and overall survival

Characteristics	Univariate Cox HR [95% CI]	*p*	Multivariate Cox HR [95% CI]	*p*
Progression-free survival				
SUV_max_	1.11 [1.05–1.16]	**<0.001**	1.12 [1.04–1.21]	**0.003**
ECOG PS 1&2 vs 0	2.03 [1.22–3.40]	**0.007**	1.47 [0.72–3.00]	0.296
De novo vs recurrent	0.57 [0.35–0.93]	**0.024**	0.55 [0.23–1.30]	0.174
Prim. endocr. resist. vs no prim endocr. resist.	2.60 [1.35–4.99]	**0.004**	1.19 [0.29–4.98]	0.807
No. of metastases: 6–10 mets vs 0–5 mets	1.70 [0.77–3.74] 1.86 [1.01–3.43]	0.191 **0.047**	2.80 [0.94–8.35] 1.67 [0.70–3.95]	0.064 0.248
No. of metastases: >10 mets vs 0–5 mets				
Ki67	1.02 [1.01–1.03]	**0.002**	1.03 [1.01–1.06]	**0.001**
G1–2 vs G3	0.51 [0.24–1.05]	0.068	0.69 [0.30–1.56]	0.372
NOS vs other subtypes	0.49 [0.27–0.89]	**0.019**	0.32 [0.14–0.73]	**0.007**
Liver metastases	1.24 [0.71–2.16]	0.448		
Lung metastases	1.21 [0.71–2.08]	0.479		

Overall survival				
SUV_max_	1.10 [1.02–1.18]	**0.016**	1.06 [0.94–1.19]	0.375
ECOG PS 1–2 vs 0	2.29 [1.12–4.68]	**0.023**	4.83 [1.55–15.09]	**0.007**
De novo vs recurrent	0.63 [0.32–1.25]	0.183	1.10 [0.33–3.81]	0.873
Prim. endocr. resist. vs no prim endocr. resist.	2.23 [1.01–5.40]	**0.049**	1.73 [0.16–18.99]	0.657
No. of metastases: 6–10 mets vs 0–5 mets	1.34 [0.38–4.74]	0.652	0.95 [0.14–6.38]	0.961
No. of metastases: >10 mets vs 0–5 mets	2.23 [0.91–5.49]	0.081	3.91 [0.90–16.91]	0.068
Ki67	1.03 [1.01–1.04]	**0.004**	1.03 [1.00–1.06]	**0.026**
G1–2 vs G3	0.30 [0.13–0.73]	**0.008**	0.29 [0.09–0.80]	**0.018**
NOS vs other subtypes	0.72 [0.30–1.77]	0.476		
Liver metastases	1.16 [0.52–2.58]	0.715		
Lung metastases	0.74 [0.32–1.70]	0.477		

Other subtypes: invasive lobular carcinoma and other histologic types. Statistically significant results are in bold.

*SUV*_max_ maximum standardized uptake value, *ECOG PS* performance status according to Eastern Cooperative Oncology Group [ECOG 1 & 2 was grouped as symptomatic disease], *Prim. endocr. resist.* primary endocrine resistance, *Tx* treatment, *No. of metastases* number of distant metastases, *NOS* not otherwise specified.

### Baseline PET and early disease progression

Among the 174 patients, 3 achieved CR (1.7%), 62 PR (35.6%), and 97 SD (55.8%). Nine patients (5.2%) experienced PD at the first response assessment after 3 months of CDK4/6i treatment, and response was not assessed in 3 patients (1.7%). Baseline SUV_max_ was significantly higher in patients who experienced early disease progression compared with those who derived clinical benefit (CR + PR + SD; *p* = 0.0037). When modeled as a continuous variable, SUV_max_ was associated with increased odds of early progression, corresponding to an 18% increase in the odds of progression per 1-unit increase in SUV_max_ (OR 1.18 per unit; 95% CI 1.05–1.33; *p* = 0.007). ROC analysis further demonstrated good discriminatory ability for progression, with an AUC of 0.78 (95% CI 0.61–0.94).

## Discussion

In this study, we demonstrated that SUVmax emerged as a strong and independent prognostic biomarker in patients receiving CDK4/6i. Higher baseline metabolic activity was associated with shorter PFS, with each 1-unit increase in SUVmax corresponding to a 12% increase in the hazard of progression. Given that a 5-unit difference in SUVmax is commonly observed across metastatic lesions, this translates into nearly a doubling of risk (HR ≈ 1.8). These findings highlight a clear biological gradient captured by metabolic tumor imaging and indicate that FDG PET/CT provides prognostic information not reflected by routine clinicopathologic variables. Taken together, our results suggest that SUVmax, a simple and universally reported PET parameter, may refine baseline risk stratification in ER-positive/HER2-negative metastatic breast cancer treated with CDK4/6i.

Histologic subtype and tumor proliferation also contributed independent prognostic value. Ki67, treated as a continuous variable, provided incremental prognostic information, with each 1% increase associated with a 3% rise in risk. Importantly, the association between SUVmax and PFS persisted after adjustment for Ki-67, metastatic pattern, and performance status, underscoring that PET-derived metabolic activity captures tumor aggressiveness beyond established markers. Consistent with prior research, our study revealed lower ^18^F-FDG uptake in lobular carcinomas compared to those not otherwise specified^20,35^. Additionally, Ki67 showed a positive correlation with SUV_max_, although the association was weak.^20,36^. This suggests that metabolic activity measured by PET reflects biological features that are not fully captured by tumor proliferation alone.

Regarding the correlation between ^18^F-FDG uptake and HER2 status, results vary. While some authors reported a significant association between ^18^F-FDG uptake and HER2 oncogene expression^36^, others did not find such a clear correlation^35^. Interestingly, even within the subset of HER2-low disease, previously considered a HER2-negative entity, differential ^18^F-FDG uptake was evident^35,36^. Specifically, patients with HER2 IHC 0 tumors exhibited higher SUV_max_ compared to patients with HER2 IHC 1+ or 2+ (with negative ISH results) tumors.

Patients with de novo disease and late recurrence, characterized by a treatment-free interval exceeding 12 months from the end of any neo/adjuvant treatment, demonstrated prolonged OS compared to the entire studied population from the Monaleesa-2 trial^37^. Similarly, our study findings revealed that patients with de novo metastatic disease experienced longer PFS and OS. We focused on patients who received CDK4/6i as the initial therapy, because their PFS and OS are markedly different from those who got it in the later stage^14^.

Endocrine resistance poses a significant challenge in the treatment of ER-positive HER2-negative breast cancer^38^. Overcoming this resistance remains a critical goal in improving patient outcomes^39^. Our study findings aligned with this trend, as patients with primary endocrine resistance exhibited shorter PFS. Furthermore, baseline SUV_max_ emerged as a better predictor of PFS than primary endocrine resistance.

Sensitivity to hormonal treatment plays a critical role in the response of HR-positive HER2-negative breast cancer^40^. Various methods exist to assess ER expression heterogeneity^41^. Notably, whole-body ER expression heterogeneity evaluated using FES-PET has been shown to differentiate patients with shorter time to progression^25^. Additionally, ^18^F-FDG-PET/CT also has the potential to identify patients with a poor outcome^32^. In clinical practice, ^18^F-FDG-PET/CT can be used as an alternative to CT and bone scans^42^. However, it is essential to maintain consistency in the chosen imaging modality for disease monitoring at baseline to ensure comparability^40^. As part of this approach, additional CE-CT was also performed at baseline.

One intriguing finding from our analysis is that a straightforward baseline SUV_max_ assessment is a prognostic biomarker. This discovery holds particular significance because previously reported evaluations using various scales, such as PERCIST, whole-body MTV, whole-body TLG, and Deauville score, tend to be more time-consuming^30–34^. A study primarily involving patients undergoing endocrine therapy showed that lower baseline SUV_max_ correlated with longer PFS^31^. Remarkably, we found that even after adding CDK4/6i to the endocrine treatment baseline SUV_max_ continued to predict treatment response. This universality suggests that SUV_max_ could serve as a reliable predictor not only for endocrine therapy but also for chemotherapy^43^ and immunotherapy^44^.

Volumetric PET parameters such as MTV and TLG were not calculated in our study, which represents a limitation, as high volumetric metabolic burden may be associated with poorer prognosis in metastatic breast cancer. In addition, this study has several other limitations.

The single-center retrospective design of our study should be acknowledged as a limitation. Although international guidelines provide general recommendations for patient selection and monitoring during CDK4/6i therapy^45^, real-world implementation varies substantially between institutions. Our center is a tertiary cancer institution with immediate on-site access to advanced imaging (including PET/CT and CE- CT), biopsy procedures and histopathological evaluation, as well as multidisciplinary supportive care such as pain management, cardio-oncology consultations and pulmonary or geriatric assessment. These organizational and diagnostic advantages may influence referral pathways, imaging timing, and treatment optimization. Consequently, it is uncertain whether centers with outsourced imaging or pathology services, limited access to PET/CT, or different diagnostic workflows would achieve comparable prognostic performance of baseline PET-derived parameters. This may limit the generalizability of our findings to settings with different diagnostic infrastructure.

Furthermore, as referral for baseline PET/CT was at the discretion of the treating oncologist, the cohort may be subject to referral bias, as not all patients initiating CDK4/6i therapy underwent PET/CT prior to treatment. In some instances of extended disease-free survival, the lack of re-biopsy in the metastatic setting resulted in the absence of current histopathology results. Consequently, we did not include histopathology results from the primary radical treatment in the analysis for such cases. Multivariable models were based on complete-case analysis, which reduced the number of patients included in the PFS and OS models due to missing histopathological and proliferative markers. The reduction in sample size in the multivariable OS model, resulting from missing histopathological and proliferative markers, may have reduced the statistical power to detect independent associations with OS. Lastly, confounding factors not captured in our study may have contributed to the OS of analyzed patients. Several potentially relevant confounders were not available for most patients, including molecular biomarkers such as ESR1 and PIK3CA mutations. The retrospective design of the study further limited the completeness of these data. As such alterations may affect both tumor metabolic activity and clinical outcomes, their absence could have attenuated or strengthened the associations observed in our analysis.

The majority of patients had only baseline ^18^F-FDG-PET/CT, which means that metabolic response could not be assessed. However, our study demonstrated that baseline results alone can provide valuable prognostic information. Interestingly, approximately 1% of patients did not exhibit ^18^F-FDG uptake, but it appears that they would align better with the more favorable prognostic group. On a positive note, this study comprised the largest patient population with metastatic breast cancer treated with CDK4/6i, and the inclusion of PET/CT assessment sets it apart. These findings may optimize clinical practice with a more personalized monitoring approach.

Overall, baseline SUV_max_, a semiquantitative marker of metabolic tumor activity, showed a strong association with PFS in patients receiving CDK4/6i for ER-positive, HER2-negative metastatic breast cancer. Patients with lower SUV_max_ experienced substantially longer PFS, indicating that this routinely reported PET parameter may refine baseline prognostic stratification in this setting. Furthermore, baseline SUVmax may help identify patients at higher risk of early disease progression. Prospective studies are warranted to validate these findings, optimally within randomized trials.

## Methods

### Study design

We conducted a single-center retrospective study at Maria Sklodowska-Curie National Research Institute of Oncology, Gliwice Branch, Poland, regarding patients who received treatment with CDK4/6i between 2018 and 2023. The study was conducted in accordance with the Helsinki Declaration of 1975, revised in 2000, and received approval from our center institutional review board (approval no. KB/430-27/22, 1st March 2022). Eligible patients included those with histologically confirmed breast cancer. The criteria for patients selection were as follows: diagnosis of advanced ER-positive, HER2-negative breast cancer, treatment with CDK4/6i combined with endocrine therapy, and imaging using PET/CT performed prior to CDK4/6i commencement, with FDG uptake in neoplastic lesions. Data collected comprised demographics, baseline characteristics, the highest SUV (SUV_max_), PFS, and OS.

The primary endpoint of this study aimed to establish the association between the baseline SUV_max_ normalized by lean body mass and PFS. Meanwhile, the secondary endpoints focused on examining the relationship between the baseline SUV_max_ and OS, as well as its association with early radiologic progression

### Imaging and response evaluation

Fluorine-18-fluorodeoxyglucose positron emission tomography (^18^F-FDG-PET), in conjunction with a non-contrast-enhanced CT, was conducted at baseline. Additionally, a contrast-enhanced thorax, abdomen, and pelvis CT scan was performed at baseline and repeated every three months for response evaluation. The ^18^F-FDG-PET scans were carried out according to internal protocol, using either the Siemens Biograph mCT (Siemens Healthineers, Erlangen, Germany) or the Gemini XL (Philips Medical Systems, Eindhoven, the Netherlands). Prior to the scan, patients fasted for 6 h. Patients were intravenously given the radiotracer (^18^F-FDG), with activities ranging from 185 to 555MBq (3.7 MBq/kg). Both scanners were calibrated to the dose calibrator on which the patient-prepared activity was measured. The acquisition was started 60 min after the tracer injection. The volume of interest (VOI) was obtained in all tumors from the base of the skull to the mid-thigh level, where ^18^F-FDG accumulation was observed. The highest maximum value of SUV within the VOI, defined as SUV_max_, served as the key parameter for the study analysis. All SUV measurements were normalized to lean body mass, in accordance with quantitative ^18^F-FDG PET/CT guidelines. This approach is particularly relevant in patients with metastatic cancer, in whom substantial changes in body weight and body composition may occur over time. Diabetic patients were eligible for inclusion. In accordance with quantitative ^18^F-FDG PET/CT guidelines, pre-scan blood glucose was required to be ≤150 mg/dL^46^.

Among 321 patients treated with first-line CDK4/6i and endocrine therapy during the study period, 176 (54.8%) underwent baseline ^18^F-FDG PET/CT and were included in the analysis. Referral for PET/CT was at the discretion of the treating oncologist.

The treatment response, evaluated using CE-CT, was categorized as complete response (CR), partial response (PR), stable disease (SD), or progressive disease (PD) per RECIST 1.1 criteria^10^. PFS represented the time from CDK4/6i commencement to either disease progression or death from any cause. OS was measured from CDK4/6i commencement to the date of death from any cause.

Primary endocrine resistance was defined as relapse occurring during the first two years of adjuvant endocrine treatment, in accordance with established guidelines^29^.

### Statistical analysis

Categorical variables were summarized using frequencies and percentages. Meanwhile, continuous variables were presented as median values along with their interquartile ranges (25–75%, IQR). To assess differences between SUV_max_ and clinicopathologic parameters, we employed the Wilcoxon rank sum test. We investigated PFS and OS using the Kaplan–Meier method, with significance testing conducted via the log-rank test. 95% confidence intervals (95% CI) for the survival curves were calculated. We estimated the effects of predictors on the probability of response by logistic regression. For continuous variables, we determined cut-off thresholds using the Receiver Operator Characteristics (ROC) curve with the Youden index. Correlation between Ki67 and SUVmax was assessed using Spearman’s rank correlation coefficient. To evaluate the effects of proposed biomarkers on PFS and OS, we employed Cox proportional hazard models. Variables with *p* < 0.30 in univariate analyses were included in the multivariable Cox models. Multivariable Cox models were fitted using complete-case analysis. As histopathological subtype, Ki67 and tumor grade were not available for all patients, models including these variables were restricted to patients with complete data. Differences in SUV_max_ between patients experiencing clinical benefit (CR + PR + SD) and those with early radiologic progression (PD at first assessment) were assessed using the Wilcoxon rank-sum test, with exact *p*-values reported due to the limited number of progression events. Discriminatory ability of SUV_max_ for early progression was quantified using ROC analysis and the corresponding area under the curve (AUC). All tests were two-sided, and a *p*-value < 0.05 indicated statistical significance. All analyses were conducted with Stata Statistical software (version 19.5, StataCorp, College Station, TX, USA).

### Ethics statement

This study was conducted in accordance with the Declaration of Helsinki and was approved by the Institutional Ethics Committee of the Maria Sklodowska-Curie National Research Institute of Oncology, Gliwice Branch, Poland (approval no. KB/430-27/22, issued on 1 March 2022). Owing to the retrospective study design and the use of fully de-identified data, the requirement for informed consent was waived by the Ethics Committee.

## Data Availability

The raw data supporting the conclusions of this article will be made available by the authors upon request.
